# Risk of New-Diagnosed Atrial Fibrillation After Transient Ischemic Attack

**DOI:** 10.3389/fneur.2022.905304

**Published:** 2022-07-14

**Authors:** Francisco Purroy, Mikel Vicente-Pascual, Gloria Arque, Robert Begue, Joan Farre, Yhovany Gallego, Maria Pilar Gil-Villar, Gerard Mauri, Nuria Montalà, Cristina Pereira, Coral Torres-Querol, Daniel Vazquez-Justes

**Affiliations:** ^1^Stroke Unit, Department of Neurology, Hospital Universitari Arnau de Vilanova de Lleida, Lleida, Spain; ^2^Clinical Neurosciences Group, Institut de Recerca Biomèdica de Lleida (IRBLleida), Universitat de Lleida, Lleida, Spain; ^3^Hospital Universitari Santa Maria de Lleida, Lleida, Spain

**Keywords:** transient ischemic attack, acute ischemic stroke, atrial fibrillation, biomarkers, NT-proBNP, etiology

## Abstract

**Background:**

Transient ischemic attack (TIA) provides a unique opportunity to optimize secondary preventive treatments to avoid subsequent ischemic stroke (SIS). Although atrial fibrillation (AF) is the leading cause of cardioembolism in IS and anticoagulation prevents stroke recurrence (SR), limited data exists about the risk of new-diagnosed AF (NDAF) after TIA and the consequences of the diagnostic delay. The aim of our study was to determine this risk in a cohort of TIA patients with long-term follow-up.

**Methods:**

We carried out a prospective cohort study of 723 consecutive TIA patients from January 2006 to June 2010. Median follow-up was 6.5 (5.0–9.6) years. In a subgroup of 204 (28.2%) consecutive patients, a panel of biomarkers was assessed during the first 24 h of the onset of symptoms. Multivariate analyses were performed to find out the associated factors of NDAF. Kaplan-Meier analysis was also performed to analyzed risk of SIS.

**Results:**

NDAF was indentified in 116 (16.0%) patients: 42 (36.2%) during admission, 18 (15.5%) within first year, 29 (25%) between one and five years and 27 (23.3%) beyond 5 years. NDAF was associated with sex (female) [hazard ratio (HR) 1.61 (95% CI, 1.07- 2.41)], age [[HR 1.05 (95% CI, 1.03–1.07)], previous ischemic heart disease (IHD) [HR 1.84, (95% CI 1.15–2.97)] and cortical DWI pattern [HR 2.81 (95% CI, 1.87–4.21)]. In the Kaplan-Meier analysis, NT-proBNP ≥ 218.2 pg/ml (log-rank test *P* < 0.001) was associated with significant risk of NDAF during the first 5 years of follow-up. Patients with NDAF after admission and before 5 years of follow-up had the highest risk of SIS (*P* = 0.002).

**Conclusion:**

The risk of NDAF after TIA is clinically relevant. We identified clinical and neuroimaging factors of NDAF. In addition, NT-proBNP was related to NDAF. Our results can be used to evaluate the benefit of long-term cardiac monitoring in selected patients.

## Introduction

Transient ischemic attack (TIA) provides a unique opportunity to optimize secondary preventive treatments and avoid subsequent ischemic stroke (IS) (1). Up to 20% of ISs are preceded by a TIA (2). This proportion could be significantly decreased with appropriate early management (3). Although atrial fibrillation is the leading cause of cardioembolism in IS patients in both sexes (4, 5), it is frequently unrecognized at the time of the index event (6). Indiscriminate anticoagulation for embolic strokes of undetermined source has not proven effective in different clinical trials (7, 8). In contrast, anticoagulation prevents stroke recurrence after IS caused by AF (4). Therefore, the identification of AF after IS and TIA is critical (9). A history of previous IS or TIA is a major SR risk factor in patients with AF (10). Limited data exists about the risk of new-diagnosed AF (NDAF) after TIA (11), as early management has mainly focused on the detection and treatment of intracranial or extracranial large artery atherosclerosis (LAA), which is the main predictor of SR (12–14). The practice of ECG monitoring is less common for patients with TIA than for those with IS (15), and when TIA patients are admitted to stroke units they are monitored for significantly shorter periods than patients with IS (16). A recent meta-analysis which focused only on TIA patients from different registries evidenced a lower AF detection rate than for IS and TIA cohorts (11).

The aim of our study was to determine the risk of NDAF after TIA in a cohort of consecutive TIA patients with long-term follow-up. We describe clinical characteristics, neuroimaging features and blood-biomarker patterns related to AF occurrence in order to identify those patients who would benefit from long-term cardiac monitoring. We also include a study of the prognostic repercussions of the diagnostic delay.

## Methods

### Design and Study Population

The REGITELL registry (Registro de pacientes con ictus transitorio de Lleida [in Spanish]) methodology has been described in detail previously (17, 18). Consecutive TIA patients were included from January 2006 to June 2010 following the Strengthening the Reporting of Observational Studies in Epidemiology (STROBE) statement (19). TIA was defined as a reversible episode of neurological deficit of ischemic origin that was fully resolved within 24 h. (20) Patient management was stablished according to international guidelines (21–24). All patients underwent standard routine diagnostic work-up which included 12-channel electrocardiography (ECG), transcranial and supra-aortic ultrasound test, head computerized tomography and laboratory tests. Clinical characteristics, previous diagnosis of AF, ABCD (2) score (25) and CHA_2_DS_2_-VASc risk score were recorded (10). Any potential, but non-definitive, TIA events were defined by the presence of isolated, atypical symptoms such as: unsteadiness; diplopia; dysarthria; partial sensory deficit; and unusual cortical vision (26). All cases were reviewed by the senior neurologist (FP) and classified by atherosclerosis and small vessel disease phenotypes according to the ASCOD classification (27). Patients in whom cardiopathy was suspected underwent extended cardiological examinations. Those patients without contraindications were evaluated by cranial MRI that included diffusion-weighted imaging (DWI) sequences within 7 days of symptom onset. Presence of DWI abnormalities and patterns of acute ischemic lesions were also recorded (17, 28).

Written informed consent or assent from relatives was obtained for all the participants. The study was approved by our local Ethics Committee: the “Comité d'Etica i Investigació Clínica de l'Hospital Universitari Arnau de Vilanova de Lleida” (18).

### New-Diagnosed AF Definition and ECG Procedures

AF was defined as a period of >30 s duration of an absolute arrhythmia without detectable P-wave activity and irregular RR interval (4). The way AF was detected was recorded: ECG at emergency department, continuous electrocardiographic monitoring (CEM) or Holter ECG on admission, and ECG after clinical symptoms during the follow-up. Holter ECG was performed within the first 48 h after admission. Holter data were analyzed by a blinded cardiologist. CEM (GE Healthcare, Chicago, IL, USA) was started immediately after admission and maintained for 3 days. It included a special software for AF detection. Whenever AF was suspected, an experienced neurologist (FP, DVJ, MV or GM) reviewed the CEM report for confirmation or dismissal. When a doubt arose, the ECG was reviewed by a cardiologist. Cases of suspected abnormal baseline ECG were reviewed by the same team.

Structured clinical visits were performed by a stroke physician (FP, MBR, YG, MPGV, GM, DVJ, MVP or MP) during the follow-up period to determine the risk of further vascular events. SR was defined by the appearance of new focal symptoms or signs associated with acute ischemic changes shown in neuroimaging scans (CT or MRI) made at 7 days, 3 months,1, 5, and 10 years. If a patient moved out of the local area or if travel to the hospital was impossible, the follow-up was conducted by phone. Recurrent events and NDAF were also actively identified by an annual review of electronic medical records (18).

### Biomarkers Substudy

From January 2008 to June 2010, blood samples were obtained by standard venipuncture within the first 24 h after the onset of symptoms at the emergency department admission to test a panel of biomarkers (29) that included high-sensitivity C-reactive protein (hs-CRP), interleukin-1-alpha (IL-1 α), IL-6, tumor necrosis factor-alpha (TNF-α), neuron-specific enolase (NSE), S100b, N-terminal pro-B type natriuretic peptide (NT-proBNP), copeptin, adiponectin and neopterin. Plasma, serum and buffy coat were obtained after centrifugation at 3,000 g at 4°C for 10 min, and aliquoted into cryovials for immediate storage at −80°C (Plataforma Biobancos PT17/0015/0027). Hs-CRP, IL-6, NSE, S100b and NT-proBNP levels were assessed in plasma samples (Hoffmann-La Roche, Basel, Switzerland) by an electrochemical chemiluminescence immunoassay using the COBAS 6000 e601 (Hoffmann-La Roche, Basel, Switzerland) at the Medical Laboratory of the HUAV. Other determinations were performed at the Clinical Neurosciences laboratory of the IRBLleida (Institut de Recerca Biomedica de Lleida). In detail, IL-1 α and TNF-α were quantified in serum using a solid-phase sandwich enzyme-linked immune sorbent assay (ELISA), commercially available in BIONOVA. The absorbance was read in a spectrophotometer using 450 nm wavelength. Copeptin was measured with a chemiluminescence sandwich immunoassay, determined with an immunoluminometric assay (Thermo Scientific B.R.A.H.M.S CT-proAVP LIA). Neopterin was quantified by competitive immunoassay using high-affinity monoclonal antibody (IBL America, MN, USA), and adiponectin was quantified with a competitive enzyme immunoassay ADIPOQ (Human) ELISA kit (Abnova).

### Statistical Analysis

We compared the baseline characteristics, ASCOD classification, presence and distribution of acute lesions in DWI, ABCD^2^ score^25^, CHA_2_DS_2_-VASc^10^, outcomes and biomarker levels between non-AF, previous AF and NDAF patient groups. The quantitative variables were compared using either the student's *T*-test or the Mann-Whitney U test. The qualitative variables were compared using the chi-squared test or Fisher's exact test when the expected cell frequency was <5. Biomarkers were not normally distributed (P-P plot), and values were expressed as median (interquartile range). A Bonferroni correction (a multiple-comparison correction) was applied to all the significant associations to reduce the risk of finding false-positive associations. To calculate the sensitivity and specificity for biomarker cut-off values which allowed to discriminate TIA patients with NDAF from TIA patients without AF, a receiver operator characteristic (ROC) analysis was performed. The cumulative risks of NDAF after excluding patients with previous AF during follow-up were estimated using a Kaplan-Meier analysis. The results were censored at the time of the outcome event, patient death, or the end of the follow-up period. Risks were compared using the log-rank test. Data on patients with no information at 10 years were censored at the time of the last available follow-up. A Cox proportional hazards multivariable analysis was performed including clinical variables (model 1), clinical variables and neuroimaging features (model 2), clinical variables and biomarker data (model 3), and clinical variables, neuroimaging features and biomarker data (model 4) to identify predictors of new diagnosis of AF after TIA. It was also adjusted for patient characteristics that significantly predicted outcomes in univariate logistic regression models. We compared the risk of stroke recurrence in subgroups of patients categorized according to the main identified predictors using a Kaplan-Meier analysis and the log-rank test. All the tests were 2-sided. Missing data was included as a random effect when fitting the model for multivariable analyses. The statistical analysis of the data was carried out using the SPSS statistical package, version 24.0. (SPSS, Chicago, IL, USA) and Graphpad software version 6 (LLC).

## Results

**Figure 1:**
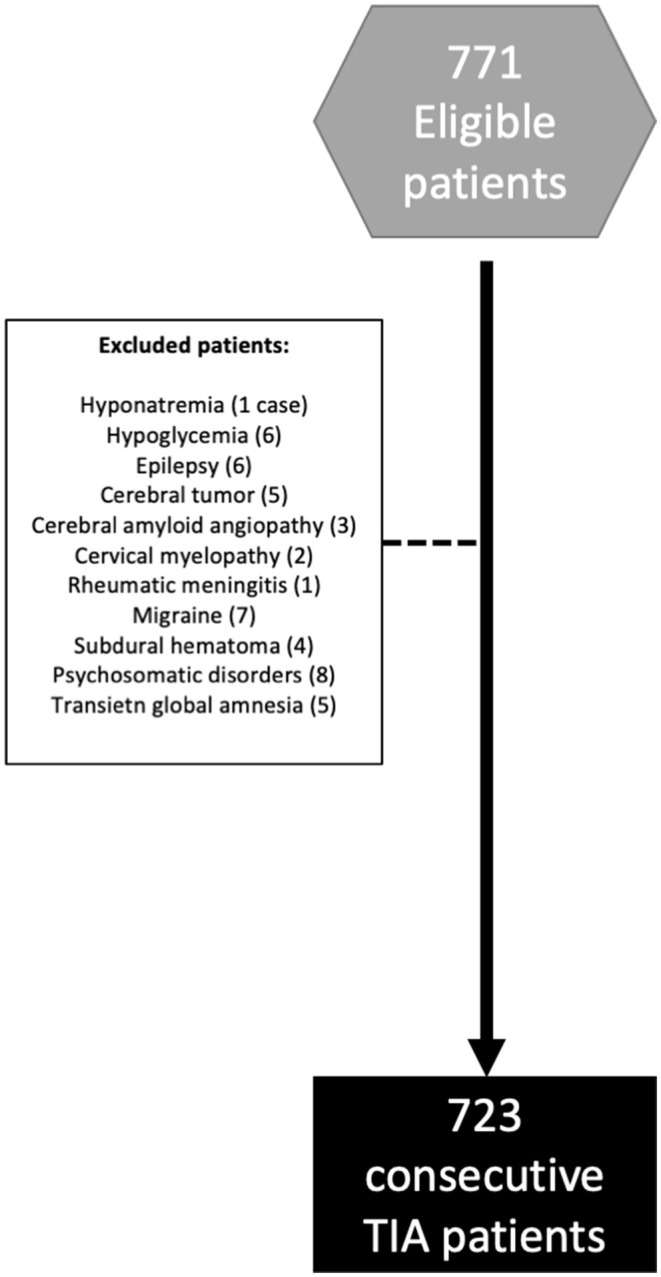
Flow chart of enrolled patients.

**Figure 2:**
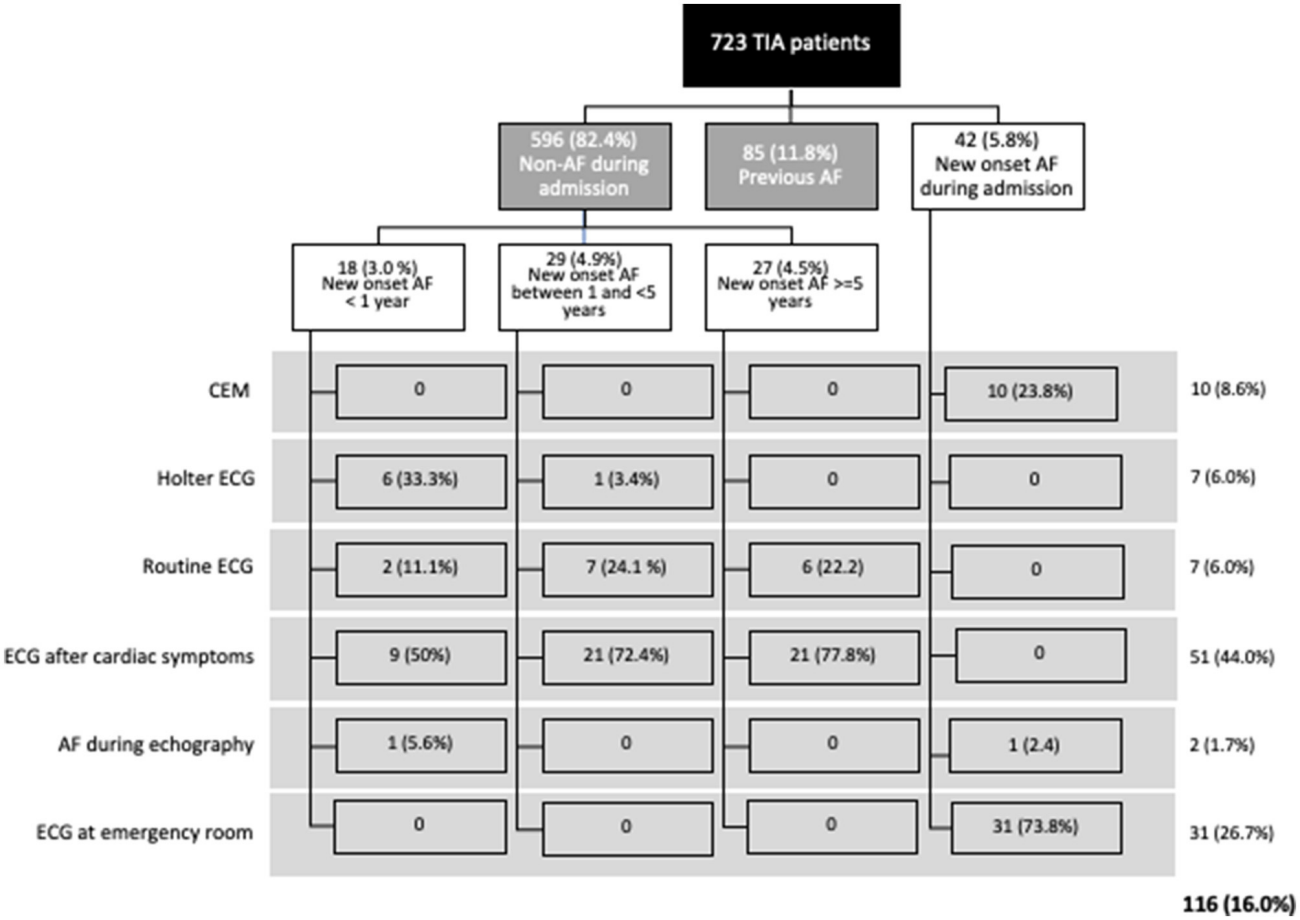
Proportion of patients with previous and new diagnosis of atrial fibrillation; and method of atrial fibrillation detection.

A total of 723 patients were included in the analysis after excluding 48 patients who were diagnosed as mimics (Figure 1). Basic demographic information are listed in Table 1. Mean age was 70.7 (SD 11.9) years. Three hundred and two patients (41.8%) were female. The median follow-up time was 6.5 (interquartile range 5.0–9.6) years. Of the 614 (84.9%) patients who underwent DWI [4.0 (SD 1.8) days after the index event], acute ischemic lesions were identified in 244 (39.7%). Previous AF was presented in 85 (11.8) subjects. According to Figure 2, NDAF after TIA was diagnosed in 116 (16.0%) patients: 31 (26.7%) of them after performing an ECG at the emergency room and 11 (9.5%) during admission, 18 (15.5%) after admission and during the first year of the follow-up, 29 (25.0%) between 1 and 5 years and 27 (23.3%) beyond 5 years of follow-up (Figure 2). 204 (28.2%) patients were included in the biomarker substudy (Supplementary Table 1).

**Table 1:** Clinical characteristics, neuroimaging features and outcomes of the TIA cohort.

	**All**
Total, *n* (%)	723
Years of follow-up, median (IQR)	6.5 (5.0–9.6)
Sex female, *n* (%)	302 (41.8)
Age, mean (SD) years	70.7 (11.9)
Previous ischemic stroke *n* (%)	66 (9.1)
Hypertension	481 (66.5)
Alcoholism, *n* (%)	21 (2.9)
Previous ischemic heart disease, *n* (%)	99 (13.7)
Diabetes mellitus, *n* (%)	215 (29.7)
Active smoking, *n* (%)	102 (14.1)
Previous atrial fibrillation, *n* (%)	85 (11.8)
Previous peripheral artery disease, *n* (%)	26 (3.6)
Hypercholesterolemia, *n* (%)	246 (34.0)
Previous heart failure, *n* (%)	31 (4.3)
Characteristics of the event	
Systolic arterial pressure, mean (SD) mmHg	152.8 (28.2)
Diastolic arterial pressure, mean (SD) mmHg	80.0 (13.1)
Duration of the event, *n* (%)	
≤ 10'	86 (12.1)
10' to 60'	248 (34.9)
≥ 60'	377 (53.0)
Missing	12
Multiple events, *n* (%)	165 (22.8)
Carotid territory event, *n* (%)	367 (50.8)
Vertebrobasilar event, *n* (%)	78 (10.8)
Undetermined territory event, *n* (%)	286 (39.6)
Possible, not definitive TIA event, *n* (%)	79 (10.9)
Speech impairment, *n* (%)	449 (62.1)
Motor impairment, *n* (%)	377 (52.1)
Isolated sensory impairment, *n* (%)	53 (7.3)
Campimetric visual deficit, *n* (%)	22 (3.0)
ABCD2 score, median (IQR)	5.0 (4.0-6.0)
Missing	12
CHA2DS2-VASC, mean (SD)	3.0 (1.6)
ASCOD Grades, *n* (%)	
A1 or 2	144 (19.9)
A0 or 3	579 (80.1)
S1 or 2	109 (15.1)
S0 or 3	614 (84.9)
Neuroimaging features	
Positive DWI, *n* (%)	244 of 614 (39.7)

*IQR, interquartile range; SD, standard deviations; DWI, diffusion weighted imaging*.

### Basal Clinical, Imaging and Blood-Biomarkers Characteristics of Patients With New-Diagnosed Atrial Fibrillation

Patients with NDAF were older [74.6 (SD 9.9) vs. 68.9 (SD 12.4), *p* < 0.001], had a higher proportion of carotid territory events (60.3 vs. 46.4%, *p* = 0.006), speech impairment (73.3 vs. 57.9%, *p* = 0.002) and previous ischemic heart disease (23.3 vs. 10.3, *p* < 0.001), but a lower proportion of non-definitive TIA events (3.4 vs. 13.8, *p* = 0.002) and isolated sensory impairment (4.3 vs. 9.0, *p* = 0.095) than patients without AF (Table 2). The proportion of women (55.2 vs.38.3%, *p* = 0.001), the CHA_2_DS_2_-VASc score [3.4 (SD 1.6) vs. 2.7 (SD 1.7), *p* < 0.001] was higher than non-AF groups.

**Table 2:** Clinical characteristics, neuroimaging features, outcomes and biomarker level by previous and new diagnosis of atrial fibrillation.

	**All**	**No AF**	**Previous AF**	**New diagnosis of AF**	**p-value comparison between all groups**	**p-value** **comparison between no previous AF and new diagnosis of AF**	**p-value comparison between previous AF and new diagnosis of AF**
Total, *n* (%)	723	522 (72.2)	85 (11.8)	116 (16.0)			
Years of follow-up, median (IQR)	6.5 (5.0–9.6)	6.6 (5.1–9.8)	5.9 (3.3–8.1)	6.7 (5.1–9.9)	0.003	0.458	0.020
Sex female, *n* (%)	302 (41.8)	200 (38.3)	38 (44.7)	64 (55.2)	0.003*	0.001*	0.095
Age, mean (SD) years	70.7 (11.9)	68.9 (12.4)	76.8 (8.0)	74.6 (9.9)	<0.001*	<0.001*	0.143
Previous ischemic stroke *n* (%)	66 (9.1)	50 (9.6)	8 (9.4)	8 (6.9)	0.660	0.363	0.515
Hypertension	481 (66.5)	328 (62.8)	70 (82.4)	83 (71.6)	0.001*	0.076	0.076
Alcoholism, *n* (%)	21 (2.9)	18 (3.4)	1 (1.2)	2 (1.7)	0.364	0.335	1.000
Previous ischemic heart disease, *n* (%)	99 (13.7)	54 (10.3)	18 (21.2)	27 (23.3)	<0.001*	<0.001*	0.724
Diabetes mellitus, *n* (%)	215 (29.7)	158 (30.3)	26 (30.6)	31 (26.7)	0.739	0.450	0.548
Active smoking, *n* (%)	102 (14.1)	86 (16.5)	3 (3.5)	13 (11.2)	0.004	0.156	0.047
Previous peripheral artery disease, *n* (%)	26 (3.6)	24 (4.6)	0 (0)	2 (1.7)	0.054	0.157	0.224
Hypercholesterolemia, *n* (%)	246 (34.0)	170 (32.6)	31 (36.5)	45 (38.8)	0.388	0.199	0.737
Previous heart failure, *n* (%)	31 (4.3)	12 (2.3)	14 (16.5)	5 (4.3)	<0.001*	0.224	0.004
**Characteristics of the event**					
Duration of the event ≥ 60', *n* (%)	377 (53.0)	265 (51.9)	49 (57.6)	63 (54.8)	0.065	0.525	0.300
Multiple events, *n* (%)	165 (22.8)	128 (24.5)	12 (14.1)	25 (21.6)	0.099	0.498	0.179
Carotid territory event, *n* (%)	367 (50.8)	242 (46.4)	55 (64.7)	70 (60.3)	0.001*	0.006	0.529
Vertebrobasilar event, *n* (%)	78 (10.8)	58 (11.1)	11 (12.9)	9 (7.8)	0.456	0.287	0.225
Undetermined territory event, *n* (%)	286 (39.6)	228 (43.7)	21 (24.7)	37 (31.9)	0.001*	0.020	0.266
Possible, non-definitive TIA event, *n* (%)	79 (10.9)	72 (13.8)	3 (3.5)	4 (3.4)	<0.001*	0.002*	0.975
Speech impairment, *n* (%)	449 (62.1)	302 (57.9)	62 (72.9)	85 (73.3)	0.001*	0.002*	0.958
Motor impairment, *n* (%)	377 (52.1)	271 (51.9)	44 (51.8)	62 (53.4)	0.954	0.765	0.813
Isolated sensory impairment, *n* (%)	53 (7.3)	47 (9.0)	1 (1.2)	5 (4.3)	0.015	0.095	0.197
Campimetric visual deficit, *n* (%)	22 (3.0)	12 (2.3)	5 (5.9)	5 (4.3)	0.141	0.225	0.613
ABCD^2^ score, median (IQR)	5.0 (4.0–6.0)	5.0 (4.0–6.0)	5.0 (4.0–6.0)	5.0 (4.0–6.0)	0.500	0.840	0.616
Missing	12						
CHA_2_DS_2_-VASc, mean (SD)	3.0 (1.6)	2.7 (1.7)	3.7 (1.3)	3.4 (1.6)	<0.001*	<0.001*	0.258
**ASCOD classification**, ***n*** **(%)**					
A1 or 2	144 (19.9)	115 (22.0)	11 (12.9)	18 (15.5)	0.065	0.118	0.608
A0 or 3	579 (80.1)	407 (78.0)	74 (87.1)	98 (84.5)			
S1 or 2	109 (15.1)	100 (19.2)	2 (2.4)	7 (6.0)	<0.001*	0.001*	0.212
S0 or 3	614 (84.9)	422 (80.8)	83 (97.6)	109 (94.0)			
Neuroimaging features							
Positive DWI, *n* (%)	244 (39.7)	158 (34.9)	28 (43.8)	58 (59.8)	<0.001_*_	<0.001*	0.046
Missing	109						
**Pattern of DWI lesion**, ***n*** **(%)**					
Scattered in one vascular territory	92 (37.6)	53 (33.5)	17 (60.7)	22 (37.3)	0.024	0.606	0.040
Cortical lesion in one vascular territory	73 (29.8)	43 (27.2)	6 (21.4)	24 (40.7)	0.082	0.056	0.078
Multiple vascular territories	19 (7.8)	12 (7.6)	2 (7.1)	5 (8.5)	0.969	0.830	0.831
Subcortical	61 (24.9)	50 (31.6)	3 (10.7)	8 (13.6)	0.004	0.007	0.709
Follow-up events							
Ischemic stroke recurrence, *n* (%)	98 (13.6)	65 (12.5)	12 (14.1)	21 (18.1)	0.271	0.107	0.451
**Biomarker results; median (IQR)**					
NSE, pg/ml	7.3 (5.0–10.0)	6.9 (4.8–9.7)	6.7 (4.9–11.0)	7.5 (5.0–10.3)	0.146	0.069	0.914
Hs-CRP, mg/l	3.6 (1.5–9.6)	3.2 (1.4–9.3)	5.2 (1.9–17.7)	2.7 (1.3–13.0)	0.228	0.970	0.182
IL-6, pg/ml	3.9 (1.8–8.5)	3.4 (1.5–7.5)	5.5 (1.9–10.1)	4.6 2.0–7.3	0.119	0.517	0.228
NT-proBNP, pg/ml	227.3 (76.5–561.8)	161.3 (57.2–377.9)	1416.0 (510.4–2507.5)	466.4 (218.7–1031.0)	<0.001*	<0.001*	0.009
Neopterin, nmol/l	13.4 (8.4–21.5)	12.4 (8.1–17.4)	8.6 (6.0–15.5)	11.9 (9.6–20.2)	0.493	0.550	0.254
Copeptin, pmol/l	9.8 (6.5–17.6)	8.4 (5.6–15.1)	11.7 (6.5–28.7)	9.4 (5.8–10.5)	0.260	0.288	0.405
Adiponectin, microg/ml	10.3 (7.1–14.3)	8.6 (6.0–12.9)	11.5 (8.5–18.3)	11.7 (8.2–17.8)	0.004	0.030	0.433
TNF-α, pg/ml	87.0 (63.5–144.5)	81.0 (62.3–132.8)	131.0 (55.0–147.5)	93.0 (69.0–151.0)	0.578	0.288	0.595
IL-1 α, pg/ml	98.0 (68.0–161.0)	90.5 (67.3–150.0)	146.0 (64.5–166.0)	109.0 (75.0–169.0)	0.498	0.224	0.746
S100b, pg/ml	36.9 (26.5–52.7)	34.5 (25.6–49.9)	40.9 (23.8)	31.8 (23.7–65.1)	0.541	0.963	0.299

*IQR, interquartile range; SD, standard deviation; DWI, diffusion-weighted imaging; ASCOD (A, atherosclerosis; S, small vessel disease; C, cardiac pathology; O, other causes; and D, dissection) classification. hs-CRP, high-sensitivity C-reactive protein; IL, interleukin; TNF-α, tumor necrosis factor-alpha; NSE, neuron-specific enolase; NT-proBNP, N-terminal pro-B type natriuretic peptide.*

* *Statistical significance after Bonferroni adjustment*.

Positive DWI (59.8 vs. 34.9%, *p* < 0.001) and cortical DWI patterns (40.7 vs. 27.2%, *p* = 0.056) were higher in new diagnosed AF than in non-AF patient group.

New diagnosed AF patients had higher levels of NT-proBNP [median (IQR) 466.4 [218.7–1031.0) vs. 161.3 (57.2–377.9) pg/mL, *p* = 0.009] and adiponectin [11.7 (8.2–17.8) vs. 8.6 (6.0–12.9) mg/mL, p = 0.030] than non AF patients.

### Predictors of New-Diagnosed Atrial Fibrillation

NDAF was associated with sex (female) [hazard ratio (HR) 1.61 (95% CI, 1.07–2.41); *P* = 0.013], age [HR 1.05 (95% CI, 1.03–1.07); *P* < 0.001], previous ischemic heart disease [HR 1.84 (95% CI 1.15–2.97); *P* = 0.012], and cortical DWI lesion pattern [HR 2.81 (95% CI 1.87–4.21); *P* < 0.001] (Table 3). A cut-off value was obtained for NT-proBNP of 218.3 pg/ml, with a sensitivity of 80.6 % and a specificity of 60.5%.

**Table 3:** Cox proportional hazards regression model to assess risk of atrial fibrillation after TIA.

	**Model 1**		**Model 2**		**Model 3**		**Model 4**	
**Variable**	**HR (95% CI)**	***P*-value**	**HR (95% CI)**	***P*-value**	**HR (95% CI)**	***P*-value**	**HR (95% CI)**	***P*-value**
Sex female	1.60 (1.10–2.31)	0.013	1.61 (1.07–2.41)	0.022				
Age	1.05 (1.03–1.07)	<0.001	1.05 (1.03–1.08)	<0.001	1.07 (1.01–1.13)	0.028	1.06 (1.00–1.13)	0.042
Previous IHD	2.04 (1.32–3.16)	0.001	1.84 (1.15–2.97)	0.012				
Carotid territory event	1.45 (0.98–2.14)	0.064	1.88 (1.23–2.87)	0.003				
S0 or 3 ASCOD classification	2.80 (1.27–6.14)	0.010			5.19 (0.70–38.50)	0.107	5.10 (0.69–37.80)	0.111
Non-subcortical DWI pattern			2.81 (1.87–4.21)	<0.001				
NT proBNP>218.2 pg/ml					4.20 (1.51–11.67)	0.006	4.10 (1.46–11.51)	0.007

*DWI, diffusion-weighted imaging; HR, hazard ratio; IHD, ischemic heart disease; ASCOD (A, atherosclerosis; S, small vessel disease; C, cardiac pathology; O, other causes; and D, dissection) classification*.

**Figure 3:**
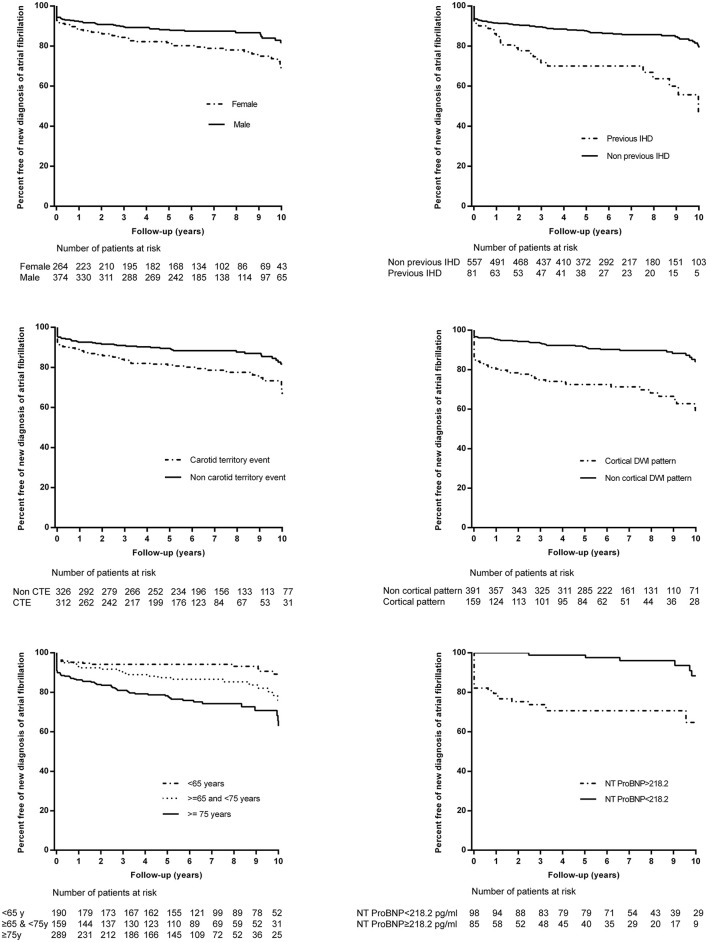
Kaplan-Meier event curves for the risk of new diagnosis of atrial fibrillation (AF) among patients with no previously diagnosed AF according to sex, previous ischemic heart disease (IHD), age, carotid territory event (CTE), diffusion-weighted imaging (DWI) pattern and NT-proBNP levels.

In the Kaplan-Meier analysis (Figure 3), female patients (log-rank test *P* = 0.002), patients with previous ischemic heart disease (IHD) (log-rank test *P* =< 0.001), patients with carotid territory events (log-rank test *P* = 0.003), and patients ≥ 75 years of age (log-rank test *P* < 0.001) had a significantly higher risk of NDAF throughout follow-up. In contrast, cortical DWI lesion patterns (log-rank test *P* < 0.001) and NT-proBNP ≥ 218.2 pg/ml (log-rank test *P* < 0.001) were associated with a significant risk of NDAF during the early follow-up, especially during the first 3–5 years.

### Comparation of the Different Groups of AF Patients

When we compared previous and NDAF groups we detected a higher proportion of women in the NDAF group [38 (44.7%) vs. 64 (55.2%); *P* = 0.095], of previous heart failure [14 (16.5%) vs. 5 (4.3%); *P* = 0.004] and of positive DWI [28 (43.8%) vs. 58 (59.8%); *P* < 0.001] (Table 2). Hypertension was more frequent in previous AF patients [70 (82.4%) vs. 83 (71.6%); *P* = 0.095]. Finally, lesions on DWI classified as scattered in one vascular territory pattern were more frequent in the previous AF group than in the NDAF group [17 (60.7%) vs. 22 (37.3%); *P* = 0.040]. In contrast, cortical lesion in one vascular territory was present in a greater proportion in NDAF patients [6 (21.4%) vs. 24 (40.7%); *P* = 0.078.

When we analyzed the different categories of NDAF with respect to time of diagnosis from the onset of symptoms (Table 4), we identified a higher proportion of patients with cortical DWI pattern and S1 or S2 ASCOD grades in patients with NDAF detected ≥ 5 years after the onset of symptoms. In addition, these patients had lower levels of NT-proBNP and copeptin.

**Table 4:** Clinical characteristics, neuroimaging features, outcomes and biomarker levels in new-diagnosed atrial fibrillation groups by time to diagnosis.

	**New diagnosis of AF**	**New diagnosis of AF during admission**	**New diagnosis of AF admission <1 year**	**New diagnosis of AF 1- <5 years**	**New diagnosis of AF ≥ 5years**	**P value**
Total, *n* (%)	116 (16.0)	42 (36.2)	18 (15.5)	29 (25.0)	27 (23.3)	
Years of follow-up, median (IQR)	6.7 (5.1–9.9)					
Sex female, *n* (%)	64 (55.2)	21 (50.0)	10 (55.6)	16 (55.2)	17 (63.0)	0.773
Age, mean (SD) years	74.6 (9.9)	74.6 (10.4)	77.2 (11.4)	75.4 (8.7)	72.0 (9.1)	0.357
Previous ischemic stroke n (%)	8 (6.9)	4 (9.5)	0 (0)	2 (6.9)	2 (7.4)	0.616
Hypertension	83 (71.6)	29 (71.6)	14 (77.8)	20 (69.0)	20 (74.1)	0.884
Alcoholism, *n* (%)	2 (1.7)	0 (0)	0 (0)	0 (0)	2 (7.4)	0.082
Previous ischemic heart disease, *n* (%)	27 (23.3)	7 (16.7)	5 (27.8)	10 (34.5)	5 (18.5)	0.306
Diabetes mellitus, *n* (%)	31 (26.7)	7 (16.7)	8 (44.4)	11 (37.9)	5 (18.5)	0.049
Active smoking, *n* (%)	13 (11.2)	3 (7.1)	2 (11.1)	2 (6.9)	6 (22.2)	0.210
Previous peripheral artery disease, *n* (%)	2 (1.7)	0 (0)	1 (5.6)	1 (3.4)	0 (0)	0.351
Hypercholesterolemia, *n* (%)	45 (38.8)	17 (40.5)	5 (27.8)	15 (51.7)	8 (29.6)	0.265
Previous heart failure, *n* (%)	5 (4.3)	1 (2.4)	2 (11.1)	1 (3.4)	1 (3.7)	0.480
**Characteristics of the event**						
Duration of the event ≥ 60', *n* (%)	63 (54.8)	21 (51.2)	8 (44.4)	18 (62.1)	16 (59.3)	0.582
Multiple events, *n* (%)	25 (21.6)	10 (23.8)	4 (22.2)	5 (17.2)	6 (22.2)	0.928
Carotid territory event, *n* (%)	70 (60.3)	27 (64.3)	9 (50.0)	20 (69.0)	14 (51.9)	0.425
Vertebrobasilar event, *n* (%)	9 (7.8)	2 (4.8)	2 (11.1)	1 (3.4)	4 (14.8)	0.328
Undetermined territory event, *n* (%)	37 (31.9)	7 (38.9)	8 (27.6)	8 (27.6)	9 (23.3)	0.874
Possible, non-definitive TIA event, *n* (%)	4 (3.4)	1 (2.4)	1 (5.6)	1 (3.4)	1 (3.7)	0.942
Speech impairment, *n* (%)	85 (73.3)	32 (76.2)	13 (72.2)	21 (72.4)	19 (70.4)	0.956
Motor impairment, *n* (%)	62 (53.4)	20 (47.6)	12 (66.7)	17 (58.6)	13 (21.0)	0.484
Isolated sensory impairment, *n* (%)	5 (4.3)	3 (7.1)	0 (0)	1 (3.7)	0.930	0.636
Campimetric visual deficit, *n* (%)	5 (4.3)	1 (2.4)	2 (11.1)	1 (3.4)	1 (3.7)	0.480
ABCD^2^ score, median (IQR)	5.0 (4.0–6.0)	5.0 (4.0–6.0)	5.0 (4.0–6.0)	5.0 (4.0–6.0)	5.0 (4.0–6.0)	0.414
Missing	1					
CHA_2_DS_2_-VASc, mean (SD)	3.4 (1.6)	3.2 (1.6)	3.8 (1.7)	3.7 (1.3)	3.1 (1.5)	0.326
**ASCOD classification**, ***n*** **(%)**						
A1 or 2	18 (15.5)	3 (7.1)	4 (22.2)	6 (20.7)	5 (18.5)	0.303
S1 or 2	7 (6.0)	0 (0)	1 (5.6)	2 (6.9)	4 (14.8)	0.093
**Neuroimaging features**						
Positive DWI, *n* (%)	58 (59.8)	22 (61.1)	9 (64.3)	12 (50.0)	15 (65.2)	0.710
Missing	19					
**Pattern of DWI lesion**, ***n*** **(%)**						
Scattered in one vascular territory	22 (37.3)	9 (39.1)	5 (55.6)	4 (33.3)	4 (26.7)	0.548
Cortical lesion in one vascular territory	24 (40.7)	11 (47.8)	2 (22.2)	6 (50.0)	5 (33.3)	0.471
Multiple vascular territories	5 (8.5)	3 (13.0)	1 (11.1)	1 (8.3)	0 (0)	0.554
Subcortical	8 (13.6)	0 (0)	1 (11.1)	1 (8.3)	6 (40.0)	0.005
**Follow-up events**						
Ischemic stroke recurrence, *n* (%)	21 (18.1)	4 (9.5)	4 (22.2)	10 (34.5)	3 (11.1)	0.038
**Biomarker results; median (IQR)**						
NSE, pg/ml	7.5 (5.0–10.3)	7.5 (5.0–10.2)	7.9 (5.4–10.5)	6.5 (3.7–9.4)	7.1 (4.5–9.4)	0.876
Hs-CRP, mg/l	2.7 (1.3–13.0)	3.0 (1.3–10.9)	7.3 (1.7–10.9)	4.7 (1.1–9.2)	1.7 (0.8–5.1)	0.243
IL-6, pg/ml	4.6 (2.0–7.3)	4.6 (2.6–6.3)	6.8 (1.5–12.1)	3.3 (2.8–6.4)	3.7 (1.7–12.3)	0.361
NT-proBNP, pg/ml	466.4 (218.7–1031.0)	602.7 (424.6–1885.5)	602.7 (382.8–1861.0)	614.2 (197.4–1031.0)	211.8 (202.3–263.6)	0.006
Neopterin, nmol/l	11.9 (9.6–20.2)	13.9 (9.2–19.5)	12.0 (6.0–17.9)	24.6 (10.0–39.2)	11.1 (10.3–19.6)	0.400
Copeptin, pmol/l	9.4 (5.8–10.5)	10.5 (8.0–15.6)	9.9 (9.3–10.5)	8.9 (8.3–9.4)	5.3 (3.9–7.8)	0.150
Adiponectin, microg/ml	11.7 (8.2–17.8)	11.5 (8.0–19.6)	12.0 (11.7–12.3)	12.4 (12.1–12.6)	8.9 (8.0–23.0)	0.858
TNF-α, pg/ml	93.0 (69.0–151.0)	113.0 (84.5–146.5)	71.0 (49.0–93.0)	116.5 (59.0–174.0)	94.5 (71.5–145.5)	0.587
IL-1 α, pg/ml	109.0 (75.0–169.0)	119.0 (100.5–160.0)	81.5 (57.0–106.0)	130.5 (70.0–191.0)	93.5 (69.0–152.0)	0.577
S100b, pg/ml	31.8 (23.7–65.1)	31.8 (27.2–57.1)	21.4 (16.7–26.0)	24.1 (23.7–24.6)	52.8 (36.1–81.3)	0.556

*IQR, interquartile range; SD, standard deviation; DWI, diffusion-weighted imaging; ASCOD (A, atherosclerosis; S, small vessel disease; C, cardiac pathology; O, other causes; and D, dissection) classification. hs-CRP, high-sensitivity C-reactive protein; IL, interleukin; TNF-α, tumor necrosis factor-alpha; NSE, neuron-specific enolase; NT-proBNP, N-terminal pro-B type natriuretic peptide*.

### New-Diagnosed AF and Risk of Recurrent Stroke

**Figure 4:**
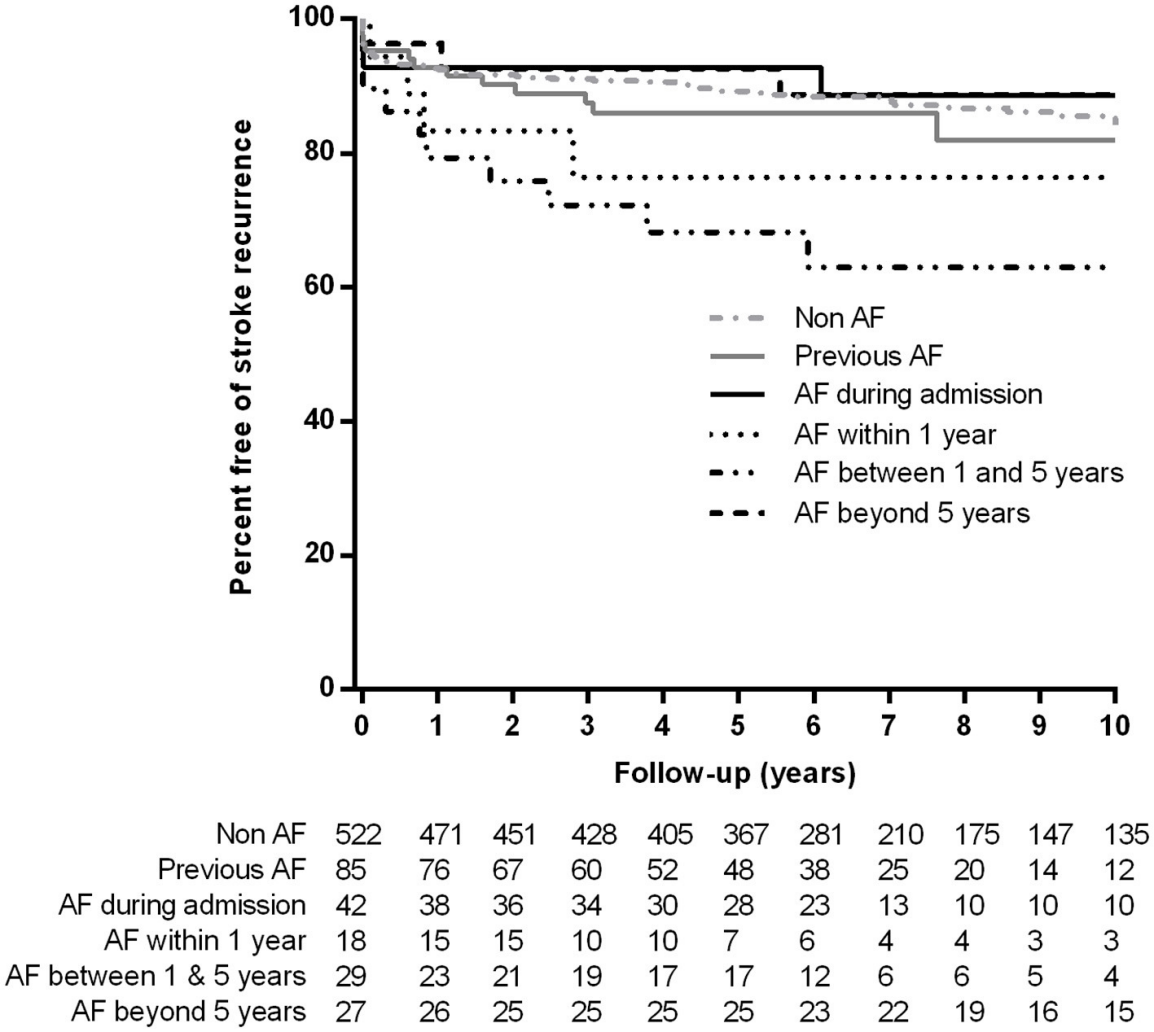
Kaplan-Meier event curves for the risk of stroke recurrence according to previous or new diagnosis of atrial fibrillation.

Although globally, no significant difference was observed in the risk of stroke recurrence between new diagnosis AF and non AF groups, patients with a new diagnosis of AF after admission and before 5 years of follow-up had a higher risk of stroke recurrence than patients with NDAF beyond 5 years of follow-up or during admission and then patients with previous AF or without AF (log-rank test *P* = 0.002) (Figure 4).

## Discussion

In our study of consecutive TIA patients attended at an emergency department, we observed a non-negligible risk of new diagnosis of AF during the long follow-up. Nearly one out of five patients without previous diagnosis of AF developed a new-diagnosed AF. TIA provides an opportunity to change the natural evolution of cerebrovascular disease. The early diagnosis of AF facilitates the start of effective treatments for secundaru prevention such as anticoagulation (30). However, the identification of paroxysmal AF after IS or TIA is a challenge. Interestingly, in our long follow-up study we identified clinical, neuroimaging and blood-biomarker predictors of NDAF that could be used to indicate patients who would benefit from long-term ECG monitoring. Some of these predictors differed from clinical and neuroimaging features of previous AF patients. In addition, patients with a new diagnosis of AF beyond 5 years of follow-up differed in the etiological phenotype of the index event and in the pattern of biomarker levels from early diagnosis AF-groups.

Our documented risk of AF was significantly higher than the risk observed in a previous meta-analysis that included studies with a limited follow-up (11), but similar to the multicenter TIA registry.org project that reached 5 years of follow-up. Although AF-related brain ischemic events have been associated with disability strokes (5, 31), our results suggest the need to investigate occult AF in TIA patients with suspicious embolic events. This is especially so when we take into account that delayed diagnosis is related to an increased risk of SR as correct preventive strategies are also delayed. As in previous studies, we observed that the risk of new diagnosis of AF was related to age (31–35), previous IHD (35) and DWI patterns (36). We also identified sex differences in the proportion of new-diagnosed AF. AF was more frequent among females (37) as the proportion of vascular risk factors is lower than in men (18). Patients with previously described predictors and high levels of NT-ProBNP in the absence of evidence of small vessel disease would clearly benefit from exhaustive ECG monitoring. The correlation between AF and high levels of this biomarker is well known among stroke and TIA patients (29, 38). This correlation is explained by the association between pro-BNP and atrial dilatation (38). In this regard, it should be noted that in our study patients with previous AF had higher levels of NT-proBNP than patients with new-diagnosed AF. This should be taken into account when calculating the cut-off levels in patients with suspected cardioembolic events.

Our study has some relevant limitations. First, the registry was designed with SR and not the diagnosis of new AF as the main endpoint. In this sense, ECG and echocardiographic abnormalities of atrial myopathy like left atrial enlargement or P-wave abnormalities (39, 40) were not registered. Second, a larger sample size would have better guaranteed the extrapolation of our results. Third, although there were no significant differences between patients included in the biomarker substudy and patients not included, it would have been interesting to have that information for all the patients. Finally, it is difficult to define the relevance of NDAF beyond 5 years of follow-up. In that cases, we could not be sure if the NDAF was related to the index event or not.

In conclusion, the risk of new diagnosis of AF after TIA is clinically relevant. Old age, sex-female, previous IHD, carotid territory symptoms, cortical DWI pattern lesion, absence of evidence of small vessel disease and high levels of NT-ProBNP increase the likelihood of new AF. Our results can be used to evaluate the benefit of long-term cardiac monitoring in selected patients.

## Data Availability Statement

Requests for access to the data reported in this article will be considered by the corresponding author.

## Ethics Statement

The studies involving human participants were reviewed and approved by Comité d'Etica i Investigació Clínica de l'Hospital Universitari Arnau de Vilanova de Lleida. The patients/participants provided their written informed consent to participate in this study.

## Author Contributions

MV-P, YG, MG-V, GM, NM, and DV-J: patients' recruitment and clinical data acquisition. RB: neuroimaging acquisition. GA, CP, JF, CT-Q, and FP: sample processing. FP and MV-P: data analysis. FP: conceived the study, procured funding, and wrote the paper. All authors commented on and approved submission of this manuscript.

## Funding

This study was supported by the Catalan Autonomous Government's *Agència de Gestió d'Ajuts Universitaris i de Recerca* (2017 *suport a les activitats dels grups de recerca* 1628) and the Instituto de Salud Carlos III, (08/1398, 11/02033, and 14/01574) and the INVICTUS plus Research Network.

## Conflict of Interest

The authors declare that the research was conducted in the absence of any commercial or financial relationships that could be construed as a potential conflict of interest.

## Publisher's Note

All claims expressed in this article are solely those of the authors and do not necessarily represent those of their affiliated organizations, or those of the publisher, the editors and the reviewers. Any product that may be evaluated in this article, or claim that may be made by its manufacturer, is not guaranteed or endorsed by the publisher.
